# Pilot study of placental tissue collection, processing, and measurement procedures for large scale assessment of placental inflammation

**DOI:** 10.1371/journal.pone.0197039

**Published:** 2018-05-11

**Authors:** Lindsey A. Sjaarda, Katherine A. Ahrens, Daniel L. Kuhr, Tiffany L. Holland, Ukpebo R. Omosigho, Brian T. Steffen, Natalie L. Weir, Hannah K. Tollman, Robert M. Silver, Michael Y. Tsai, Enrique F. Schisterman

**Affiliations:** 1 Division of Intramural Population Health Research, *Eunice Kennedy Shriver* National Institute of Child Health and Human Development, National Institutes of Health, Bethesda, MD, United States of America; 2 Department of Laboratory Medicine and Pathology, University of Minnesota, Minneapolis, MN, United States of America; 3 Department of Obstetrics and Gynecology, University of Utah and Intermountain Healthcare, Salt Lake City, UT, United States of America; Faculty of Animal Sciences and Food Engineering, University of São Paulo, BRAZIL

## Abstract

**Background:**

Placental dysfunction is related to many pregnancy complications, but collecting placental specimens for investigation in large scale epidemiologic studies is often infeasible. Standard procedures involving immediate collection after birth and snap freezing are often cost prohibitive. We aimed to collect pilot data regarding the feasibility and precision of a simpler approach, the collection of tissue samples following 24 hours of refrigeration of whole placentae at 4°C, as compared to the “gold standard” of snap freezing excised tissue within 40 minutes of delivery for the assessment of inflammatory cytokines.

**Methods:**

Placentae were collected from 12 women after delivering live-born singleton babies via uncomplicated vaginal delivery. Two placentae were utilized to establish laboratory tissue processing and assay protocols. The other 10 placentae were utilized in a comparison of three tissue collection conditions. Specifically, key inflammatory cytokines were measured in 3 sections, representing three collection conditions. Sections 1 (full thickness) and 2 (excised prior to freezing) were obtained within 40 minutes of delivery and snap frozen in liquid nitrogen, and section 3 (full thickness) was obtained after refrigerating the placenta at 4°C for 24 hours.

**Results:**

IL-6, IL-10, and IL-8 all had comparable concentrations and variability overall in all three section types. Levels of tumor necrosis factor alpha (TNF-α) were too low among samples to reliably measure using immunoassay.

**Conclusions:**

Refrigeration of placentae prior to processing does not appear to compromise detection of these cytokines for purposes of large scale studies. These findings provide a framework and preliminary data for the study of inflammatory cytokines within the placenta in large scale and/or resource-limited settings.

## Introduction

Placental dysfunction plays a role in common obstetric complications, such as pregnancy loss, gestational hypertension, preeclampsia, and preterm birth [1–3]. Many of these outcomes require large sample sizes to appropriately study in clinical populations, but direct assessments in placental biospecimens is limited owing to the logistical hassle and expense of collecting a tissue that is dependent on the timing of spontaneous birth and variable lengths of labor. However, placentae are generally discarded immediately following childbirth and the collection of tissue samples poses minimal, if any, risk to patients. Indeed, examination of placental specimens in large scale studies of important pregnancy complications presents an opportunity for novel study. In particular, various inflammatory biomarkers have been linked with maternal and neonatal health complications [4]. For example, IL-10 has been associated with various placenta-mediated disorders, including fetal growth restriction, preeclampsia, recurrent pregnancy loss, and placental abruption [4]. Additionally, TNF-α is significantly elevated in women with preeclampsia [5]. Furthermore, data from the Effects of Aspirin in Gestation and Reproduction (EAGeR) trial showed improved clinical pregnancy and live birth rates, as well as lower preterm birth [6], among healthy women with a history of pregnancy loss who took low-dose aspirin (81 mg), an anti-inflammatory agent [7]. Collectively, these findings lend evidence to an important role for dysregulated inflammation in pregnancy complications and support an expanded effort to study inflammation in placental biospecimens.

Standard tissue processing for protein measurement generally requires snap freezing with liquid nitrogen or other appropriate processing immediately upon tissue harvesting; such procedures would require 24-hour availability of research personnel for specimen collection and processing for placentae of spontaneous deliveries. Given limited resources, the cost of staffing study personnel around the clock at multiple clinical sites and maintaining perishable materials such as liquid nitrogen for the duration of a lengthy large-scale study renders such practice resource intensive. Establishing valid procedures for measuring inflammatory markers in placental specimens would inform the design and inclusion of placental collection in new studies, especially large-scale studies which are necessary to study rarer outcomes like preterm birth and preeclampsia. Yet, no study has systematically assessed the impact of temporary refrigeration of placental tissue after delivery followed by delayed processing on assessment of inflammatory cytokines, an approach that would enable greater exploration of this tissue in epidemiologic studies. Therefore, the aim of the present pilot study was to compare the results of cytokine concentrations after flash-freezing within 40 minutes of delivery with refrigeration for up to 24 hours before processing and storing at -80°C without flash-freezing. Because previous studies identified inflammatory markers associated with obstetric disorders, we were particularly interested for this preliminary study in whether key inflammatory cytokines had sufficient stability to be accurately assessed after this less resource-intensive collection protocol.

## Materials and methods

### Study population

Placentae were collected from 12 healthy women with no known pregnancy complications after delivering live-born, singleton babies via uncomplicated vaginal delivery following spontaneous labor at 39–40 weeks’ gestation at the University of Utah Health Sciences Center in Salt Lake City, Utah. Phase I laboratory experiments established the optimal practices for laboratory processing of placental tissue specimens by comparing several methodologies, such as tissue homogenization techniques, buffer selection, and the necessity of a protease inhibitor using tissue from 2 of the 12 collected placentae. Phase II experiments, which incorporated the optimal laboratory practices determined in Phase I, were designed to compare different methods of placental acquisition and storage after delivery on inflammatory protein levels. These experiments were conducted on the remaining 10 placentae. All women provided written informed consent. Institutional Review Board approval was obtained for all specimens by the University of Utah as well as by the University of Minnesota, where the laboratory performing the processing and analysis was located.

### Experimental procedures

#### Tissue collection

**Fig 1:**
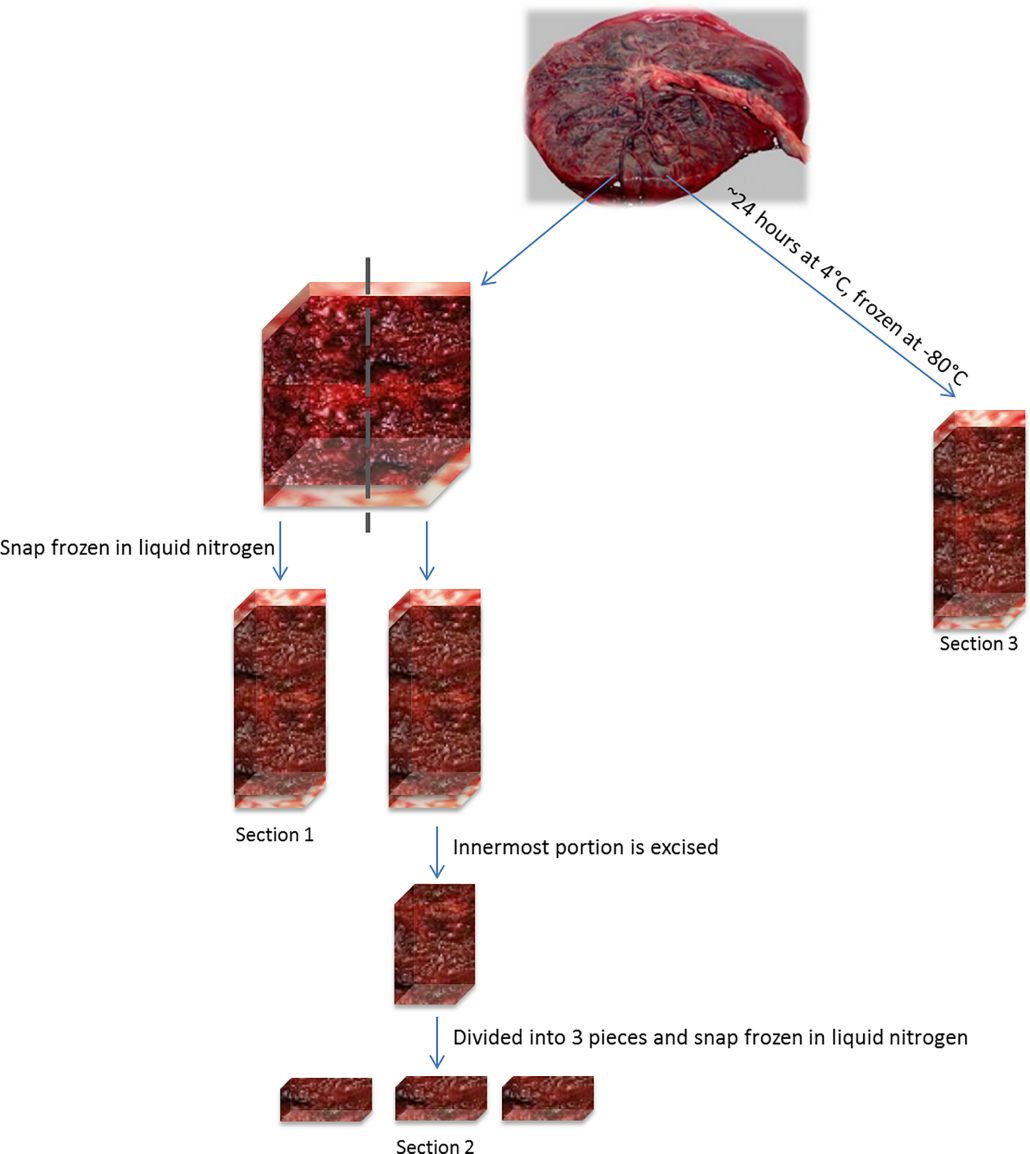
Tissue collection schematic. Tissues were collected using three different approaches: Section 1 was excised as a full thickness sample within 40 minutes of delivery and snap frozen in liquid nitrogen; Section 2 was excised within 40 minutes of delivery to the innermost portion of the placenta to reduce heterogeneity at the time of dissection and snap frozen in liquid nitrogen; Section 3 was collected 24 hours later after whole placenta refrigeration at 4°C, then was dissected as a full thickness sample and frozen at -80°C.

Within 40 minutes of vaginal placental delivery, an approximately 10 gram full-thickness “plug” of the placenta was excised using a scalpel. This tissue was then cut in half, vertically, into two approximately 5-gram plugs. One plug was placed immediately into a 15 mL cryovial and placed into liquid nitrogen (“snap frozen”) before being stored at -80°C (“section 1”). The second was dissected, with the top and bottom portions corresponding to the maternal decidua and basal/chorionic plate, respectively, discarded while the innermost tissue was divided and placed into three separate 15 mL cryovials and snap frozen in liquid nitrogen before being stored at -80°C (“section 2”). The remaining placental tissue was placed into a sealed container and refrigerated for 24 hours, at which point another full-thickness plug was removed adjacent to the previous excision, placed into a 15 mL cryovial, and stored directly into a freezer at -80°C (“section 3”). A diagram of the placental collection procedures is shown in Fig 1. All specimens were then shipped on dry ice to the designated laboratory at the University of Minnesota for analysis.

In preparation for analyte measurement, sections 1 and 3 were removed from -80°C freezers and placed on ice for 10–15 minutes to enable dissection. Using a sterile scalpel blade, sterilized forceps, and Whatman Paper, visible maternal decidua and basal/chorionic plate tissue were removed from sections 1 and 3 and the innermost tissue of these samples were placed into 5-mL cryovials and returned to storage at -80°C.

#### Phase I experimental procedures

Phase I experiments to establish optimal laboratory procedures were carried out on “section 1” samples of two placentae.

Buffer selection experiments were conducted as follows. Phosphate buffered saline (PBS), GE Mammalian Protein Extraction Buffer (MPEB), and a non-detergent sulfobetaine [Covaris, Protein Extraction Buffer SuperB (SB)] buffers were tested with a tissue to buffer ratio of 1:4. Placental samples from two placentae were bead-mill homogenized and run in up to four replicates in individual assay kits for each of the following analytes: IL-6, IL-8, IL-10 (high sensitivity kit) and TNF-α (high sensitivity kit). Additional respective buffers were used for any additional required dilutions. For IL-6, all lysates were analyzed at 3X and 12X dilutions. For IL-8, all lysates were analyzed neat (no dilution). For IL-10, all lysates were analyzed neat and at a subsequent dilution of 2X. For TNF-α, all lysates were analyzed neat and at a 3X dilution.

Protease inhibitor experiments were conducted as follows. One sample from each of 2 placentae was processed with PBS and bead-mill homogenized both with and without a protease inhibitor (cOmplete, Roche Diagnostics USA). Samples were divided in half making a total of 4 lysates. Assays were replicated without dilution four times for each of the analytes using single assay ELISA kits for IL-6 and IL-8.

Homogenization of pulverized versus intact tissue experiments were conducted as follows. Two of four tissue sections taken from both placentae were pulverized using Covaris Cryoprep at impact level 4 of 6 and the remaining two sections remained intact. Pulverized samples were either immediately homogenized and used for ELISA analysis or aliquoted into bead-filled tubes and stored at -80°C for later use. Pulverized tissue was homogenized with PBS containing protease inhibitor cocktail as previously described with a buffer to tissue ratio of 4:1. Intact tissue pieces of an approximately equal mass to the pulverized tissue were also homogenized following the same protocol. Each sample was made into four lysates and all lysates were run in four replicates for IL-6 and IL-8.

#### Phase II experimental procedures

Per procedures established in Phase I, pulverization of frozen tissues was accomplished using Covaris Cryoprep (Covaris, Woburn, USA), and three aliquots of approximately 0.625 grams powdered tissue were made from each sample, producing a total of 30 samples for analysis (10 placentae each producing three pulverized tissue samples per Section [i.e., collection condition]). Of note, only one pulverized tissue aliquot was successfully produced for further analysis from Section 3 of one placenta, thus resulting in 28 samples available for Section 3 analyses. Lysates were made from each pulverized sample using PBS with protease inhibitor and a bead mill homogenizer for 30 seconds at 5000 rpm (Minilys Bead Mill Homogenizer; Bertin Instruments; France). Samples were centrifuged at 16,000 rpm. Supernatant was collected and diluted with PBS plus protease inhibitor to reach a final 4:1 buffer to tissue ratio. Supernatant samples were placed on ice until all were available for plating.

All samples (n = 30 per collection condition) were run in replicate (n = 5) for each of the following four analytes: IL-6, IL-8, IL-10 and TNF-α. All assays were performed utilizing high sensitivity ELISA kits from R&D Systems, Minneapolis, MN. The placental lysates were used without further dilution for all assays with the exception of IL-6, which required a 3X dilution. ELISAs were performed as directed by the manufacturer, according to the protocol recommended for cell culture supernatants when available. This process was carried out three times (one time for each placental section), resulting in fifteen measurements of each analyte per placenta (45 measurements in total per placenta).

## Results

Overall, Phase I optimization experiments determined that homogenization of pulverized tissue with PBS plus protease inhibitor resulted in the best protein recovery and lowest variation (Tables 1–3); thus, this approach was applied to all subsequent procedures for Phase II tests.

**Table 1:** Cytokine concentrations across three different homogenization buffers.

	PBS	MPEB	SB
**IL-6**	5.2 (4.9,5.3)	5.8 (5.6,6.0)	5.3 (5.2,5.4)
**IL-8**	45.5 (44.3–47.1)	5.6 (4.9–5.8)	13.1 (13.0–13.2)
**IL-10**	1.2 (0.9–1.4)	7.1 (3.3,7.5)	1.1 (0.8,1.8)
**TNF-α**	0.35 (0.30,0.38)	0.22 (0.21,0.23)	0.13 (0.12,0.16)

Data are median (interquartile range) in pg/mg protein, except for IL-8 (all buffers) and IL-10 with PBS for which full data range is reported in lieu of interquartile range since only two to four values were available for each buffer. PBS, phosphate buffered saline; MPEB, GE mammalian protein extraction buffer; SB, non-detergent sulfobetaine [Covaris, Protein Extraction Buffer SuperB].

**Table 2:** Cytokine concentrations with and without protease inhibitor use during homogenization.

	Sample 1		Sample 2	
	(+) PI	(-) PI	(+) PI	(-) PI
**IL-6**	12.2 (3.2%)	11.0 (4.5%)	67.9 (2.3%)	40.3 (1.6%)
	(12.0–12.9)	(10.9–11.9)	(65.8–69.4)	(39.5–41.1)
**IL-8**	49.9 (2.9%)	36.1 (2.8%)	105.5 (3.9%)	60.6
	(48.6–51.6)	(34.4–36.8)	(102.5–108.4)	(N/A)

Data are median (% coefficient of variation)/(range) in pg/mg protein. PI, protease inhibitor.

**Table 3:** Cytokine concentrations with and without mechanical pulverization of tissue prior to homogenization.

	Sample 1		Sample 2	
	Pulverized	Intact	Pulverized	Intact
**IL-6**	3.7 (3.5,4.0)	2.6 (2.1,3.2)	76.5 (71.1,84.4)	42.5 (22.7,60.5)
**IL-8**	10.0 (9.0,10.3)	4.2 (3.4,5.0)	69.5 (62.8,74.8)	42.3 (28.9,56.9)

Data are median (25^th^, 75^th^ percentile) in pg/mg protein.

**Fig 2:**
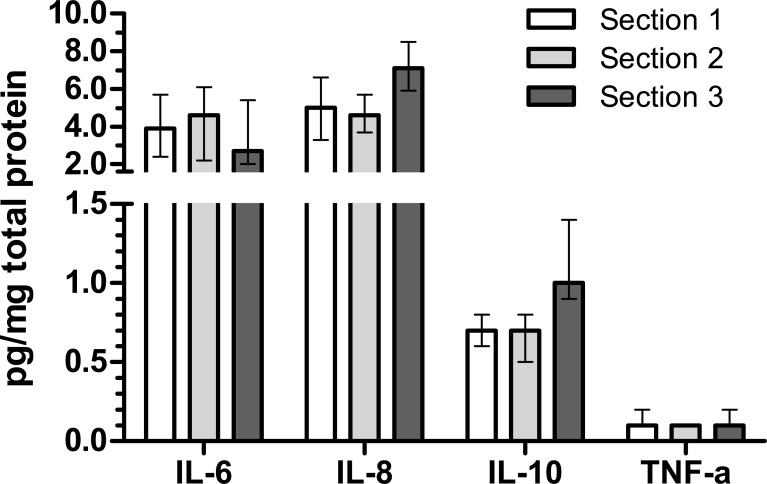
Biomarker concentrations across placental collection approaches. Data are median (25^th^, 75^th^ percentile) in pg/mg total protein. Sections 1 (full thickness) and 2 (excised middle portion prior to freezing) were obtained within 40 minutes of delivery and snap frozen in liquid nitrogen. Section 3 (full thickness) was obtained after refrigerating the placenta at 4°C for 24 hours, and placed into -80°C freezer after samples were obtained. From each of the 10 placentae used to compare collection approaches, there were three lysates produced for each collection condition, and each lysate was assayed five times. Therefore, each individual placenta contributed 15 of each cytokine measurement per collection condition.

Examining the three different placental collection conditions (Phase II), median concentrations were similar across the three sections for IL-6, IL-8, and IL-10 (Fig 2). Results for TNF-α were just above the level of detection under all conditions, limiting our ability to examine variation across collection procedures.

## Comment

Minimal variation was observed on concentrations of inflammatory cytokines in placentae stored at 4°C for 24 hours prior to processing as compared to those processed and flash-frozen within 40 minutes of delivery in the present pilot study. Additionally, we determined that mechanically pulverizing tissue during initial processing improves precision compared to using intact placental tissue. With increasing evidence of the role of inflammation in reproductive outcomes, it is crucial to consider appropriate approaches to characterize the role of placental inflammation in large scale population based or clinical studies.

Here we provide data that support tissue storage at 4°C as a valid approach that could enable greater use of often discarded placentae for measuring several inflammatory cytokines. Typically, snap freezing placental tissue prior to -80°C storage is considered the “gold standard” for tissue collection and preservation, allowing for optimal preservation of a broad range of biochemical markers and analytic approaches [8]. However, concentrations of IL-6, IL-8, and IL-10 evaluated here were similar to the gold standard approach. In agreement, previous studies report that plasma IL-6, IL-8, IL-10, and TNF-α levels remain stable when whole blood is stored at 4°C for up to 48 hours prior to separation of plasma [9]. As few similar studies with placental tissue have been conducted, information on the stability of these biomarkers in the placenta to corroborate these data is unavailable. Thus, here we provide for the first-time novel, pilot data indicating that IL-6, IL-10, and IL-8 are reasonably stable in the placenta after 24 hours of storage at 4°C.

Other measures have been taken to simplify the procurement of quality placental tissue. For example, the use of RNAlater® and DNAgard® treatments with storage at 4°C (and subsequent freezing at -80°C) preserves RNA integrity for gene expression experiments better than immediate snap-freezing with liquid nitrogen [10]. Importantly, storage in these solutions at 4°C was shown to be easier for a busy clinic and allowed tissue samples to be taken by clinicians [10]. Additionally, a previous study described refrigeration of the placenta in the event that no technician was available with processing taking place the next day, similar to the approach used in our study [11]; however, no validation for sample refrigeration was provided.

Notably, the biomarkers examined here are highly relevant to current placental study, as they are associated with adverse obstetric outcomes, including preeclampsia [12, 13]. Other relevant biomarkers were considered during the development of this pilot study but were either known to be unstable when refrigerated or required quantification techniques beyond the scope of this pilot study. For example, macrophage migration inhibitory factor (MIF) is associated with preeclampsia and fetal growth restriction (FGR), chorioamnionitis, and preterm birth; TGF-β is associated with IUGR; and RANTES is associated with preterm birth [4], but these three biomarkers were shown to be unstable in blood stored for 4 hours at 4°C [9]. Placental growth factor is stable in serum for up to 3.3 days at room temperature and at least 30 days when refrigerated [14]. Also, pregnancy-associated plasma protein A is stable in serum for 142 days at refrigerator temperature. Thus, these other biomarkers might also be of potential future interest given their role in placental function and pathology [15]. It remains important to distinguish which biomarkers may be suitable candidates for valid assay after placental refrigeration.

Lastly, pulverizing placental tissue during laboratory processing was found to improve precision compared to homogenizing intact slices of placenta. The coefficients of variation in concentrations of IL-6 and IL-8 were significantly lower in specimens cryogenically pulverized prior to homogenization. Furthermore, analyte concentrations of IL-6 and IL-8 in pulverized tissue were significantly higher compared to those that were homogenized from intact tissue. These data are not surprising since pulverization allows for greater homogeneity in tissue samples [16]. Given these observations, pulverization of placental tissue appears to be an important step for limiting variation.

There are several strengths of our study. First, the preplanned comparison of three distinct collection methods and thorough assessment of laboratory processes was novel. The placentae used were obtained from healthy women that delivered via spontaneous vaginal delivery with no known pregnancy complications, helping reduce variability for purposes of validation. Our pilot study would have benefitted from use of a prespecified random tissue sampling technique [17], as placentae are heterogeneous and cytokine concentrations may be present in higher concentrations at different locations [18]. Increasing the number of placental sections per specimen would have also strengthened the approach as one random section may not be a representative area of inflammatory activity relevant to a particular pathology or exposure of interest; such challenges will require a larger validation experiment. Our study would have further benefitted from investigating cytokines that were less stable than our chosen set, although we believe the present initial findings lay the groundwork for further study.

Overall, our findings provide a framework and preliminary data for the study of inflammatory cytokines within the placenta in large scale and/or resource-limited settings. We determined refrigeration of placenta at 4°C for up to 24 hours has minimal effect on IL-6, IL-8, and IL-10 concentrations in comparison to the gold standard of immediate collection and snap-freezing prior to storage at -80°C. Future research aimed at maximizing the use of this largely discarded, but potentially information-rich, organ would aide in a better understanding of the inflammatory markers in the placenta and their association with critical obstetric and neonatal outcomes.

## Supporting information

S1 File Raw data underlying results reported in main manuscript. (XLSX)
